# Citric Acid Water as an Alternative to Water Restriction for High-Yield Mouse Behavior

**DOI:** 10.1523/ENEURO.0230-20.2020

**Published:** 2021-02-02

**Authors:** Anne E. Urai, Valeria Aguillon-Rodriguez, Inês C. Laranjeira, Fanny Cazettes, The International Brain Laboratory, Zachary F. Mainen, Anne K. Churchland

**Affiliations:** 1 Cold Spring Harbor Laboratory, Cold Spring Harbor, NY 11724; 2 Champalimaud Centre for the Unknown, Lisbon, Portugal 1400-038; 3 Cognitive Psychology Unit, Institute of Psychology and Leiden Institute for Brain and Cognition, Leiden University, 2300 RC Leiden, The Netherlands; 4 David Geffen School of Medicine,University of California Los Angeles, Los Angeles, CA 90095

**Keywords:** citric acid water, decision-making, mouse behavior, water restriction

## Abstract

Powerful neural measurement and perturbation tools have positioned mice as an ideal species for probing the neural circuit mechanisms of cognition. Crucial to this success is the ability to motivate animals to perform specific behaviors. One successful strategy is to restrict their water intake, rewarding them with water during a behavioral task. However, water restriction requires rigorous monitoring of animals’ health and hydration status and can be challenging for some mice. We present an alternative that allows mice more control over their water intake: free home-cage access to water, made slightly sour by a small amount of citric acid (CA). In a previous study, rats with free access to CA water readily performed a behavioral task for water rewards, although completing fewer trials than under water restriction (Reinagel, 2018). We here extend this approach to mice and confirm its robustness across multiple laboratories. Mice reduced their intake of CA water while maintaining healthy weights. Continuous home-cage access to CA water only subtly impacted their willingness to perform a decision-making task, in which they were rewarded with sweetened water. When free CA water was used instead of water restriction only on weekends, learning and decision-making behavior were unaffected. CA water is thus a promising alternative to water restriction, allowing animals more control over their water intake without interfering with behavioral performance.

## Significance Statement

High-throughput, reliable behavioral training is a key requirement for the use of mice in behavioral and systems neuroscience but depends crucially on ability to motivate animals to perform specific behaviors. Here, we present an alternative method to commonly used methods of water restriction: free home-cage access to water, made slightly sour by a small amount of citric acid (CA). This non-labor-intensive, low-error option benefits animal health without hindering behavioral training progress. CA water can serve as a reliable and standardized strategy to achieve high quality task behavior, further facilitating the use of mice in high-throughput behavioral studies.

## Introduction

The mouse is an indispensable species for systems neuroscience, thanks to a rich set of available tools to record and manipulate brain structure and function, combined with the knowledge of teaching mice specific behavioral tasks. While long thought to be beyond the capacities of the mouse (Abbott, 2010), mice are now routinely trained to perform abstract sensory, navigational and decision-making tasks (Carandini and Churchland, 2013; Guo et al., 2014; Goltstein et al., 2018). Crucial to this success is the ability to motivate them to perform specific behaviors.

One successful strategy is to restrict animals’ water access, and reward them with fluids for performing behavioral tasks (Skinner, 1936; Toth and Gardiner, 2000; Guo et al., 2014; Goltstein et al., 2018). The mouse’s ability to thrive on little water matches its evolutionary past on steppes and other dry environments (Fertig and Edmonds, 1969). In a laboratory setting, however, water restriction requires rigorous monitoring of animal’s health and hydration status, usually with daily weighing and precisely measured water intake (Toth and Gardiner, 2000).

We here consider a complementary, alternative approach: free access to water, in which a small amount of citric acid (CA) has been dissolved. CA is a food preservative which makes water taste slightly sour. Rats reduce their intake of CA water without getting sick or dehydrated (Watson et al., 1986). Moreover, free CA water has only subtle impacts on rats’ willingness to perform behavioral tasks in which they are rewarded with plain water (Reinagel, 2018). This strategy has significant advantages for both animal welfare and scientific throughput. However, free access to 2% CA water led to a reduction in trial yield of around 30% in 2-h daily training sessions (Reinagel, 2018), impeding its use in high-throughput behavioral paradigms. Moreover, it is not known whether this approach would also work in mice. We here set out to test the safety and efficacy of CA water in mice, and explore further strategies that combine the benefits of freely accessible CA water with high trial yields.

Mice readily consumed CA water without adverse health effects. In adult mice, access to 2% CA water resulted in stable weights, and water intake similar to commonly used water restriction regimes. Giving mice free access to CA water on weekends did not affect trial numbers or task performance on subsequent training days. Specifically, mice with free access to CA water on weekends were motivated to perform many trials (500–1000 daily) evenly over the week, doing a decision-making task in which they earned sugar water. In a large dataset of mouse decision-making behavior (The International Brain Laboratory et al., 2020), weekend regimes of traditional water restriction versus free CA water led to similar weight and learning curves. Free access to 2% CA water thus allows animals more control over their water intake, without negative effects on behavioral performance.

## Materials and Methods

Experiments were approved by ORBEA Animal Welfare Body at the Champalimaud Center for the Unknown (CCU; number 2016/005) and the Cold Spring Harbor Laboratory (CSHL) Institutional Animal Care and Use Committee (protocol 16-13-10-7, amendment approved 2018-07-28).

### Experimental design

The data from cohort 4 are part of a large, public dataset (described in The International Brain Laboratory et al., 2020) and available at https://data.internationalbrainlab.org. All other experiments were collected separately (although in the case of cohort 3, using the same behavioral task and apparatus as described in The International Brain Laboratory et al., 2020; see also below, Decision-making task). COVID-19-related lab shutdowns and following restrictions led to unavoidable disruptions of the course of some experiments and prevented the enlargement of sample sizes.

### Cohort 1

Seventeen mice (11 male and six female wild-type Thy1-GCaMP6, C57BL/6 background, 2-3 months) were single-housed. Each animal’s baseline weight was recorded on five consecutive days, while they had free access to plain water in their home-cage. They were then divided into three groups (with males and females randomly assigned), each with its own water regime. The first group (*n* = 8) received measured amounts of water each weekday. This was either 600 μl, or 40 μl of water per gram of body weight as measured right before water administration (range 600–1150 μl). On Saturday they received double this amount, while no water was administered on Sundays. The second group (*n* = 5) had access to free water in their home-cage, in which CA (1% or 2%) was dissolved. The last group (*n* = 4) remained on free water in their home-cage and served as a control. No animals showed signs of dehydration, or were dropped from the study.

The home-cage bottles with regular or 2% CA water were weighed daily, and the weight change used as a proxy for the volume of water consumed. To measure thirst, each animal was placed in a cage with 2 ml of plain water for 5 min on days 16, 24, and 29. For the animals on measured water, these 5 min replaced their daily measured water administration.

Mice in this study were on average nine weeks old on the first day of the experimental intervention. Expected weight curves were computed from weighings of age-matched animals not undergoing fluid restriction, from The Jackson Laboratory (2015).

### Cohort 2

**Figure 1.**
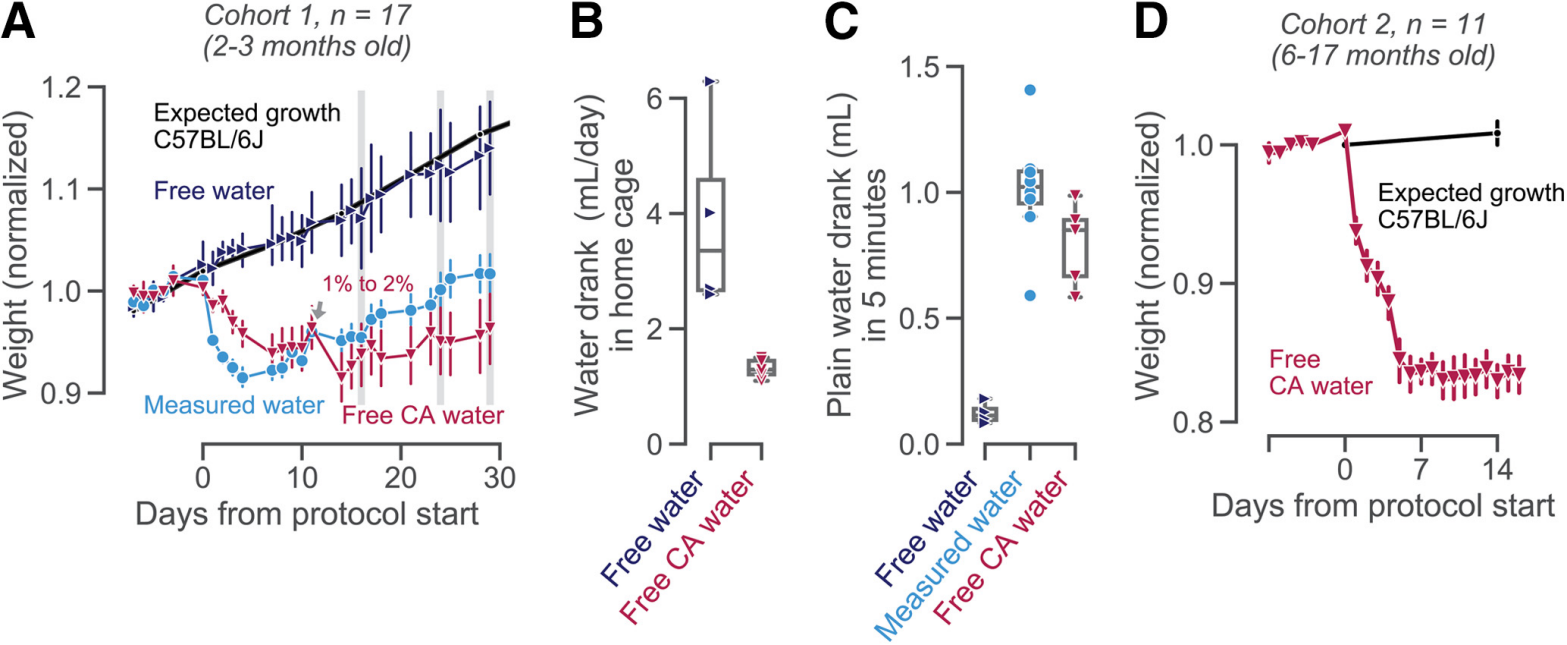
Mice maintain healthy weights and water intake on CA water. ***A***, Average weight (as a fraction of each animal’s baseline weight) for 17 young animals, divided into four experimental groups. The age-matched weight curve expected for C57BL/6J mice (The Jackson Laboratory, 2015) on free water is shown as a reference (thick black line). On day 11 (the Friday of the second week after the intervention), the CA group was switched from 1% to 2% CA in their home-cage water bottle. Error bars show mean ± 68% confidence intervals across animals. ***B***, Volume of water drank in the home-cage, estimated by measuring water bottles daily (days 11–29) for the cohorts of mice on free plain (blue) or 2% CA (red) water. Each data point is the average for one animal; boxplots show median and quartiles. ***C***, Plain water consumed in 5 min of free access, measured on days 16, 24, and 29, indicated with gray shaded bars in ***A***. Each data point is the average for one animal; boxplots show median and quartiles. ***D***, Weight curves of 11 older animals, before and after switching from free plain water to 2% CA water. Age-matched expected growth curves for C57BL/6 mice (The Jackson Laboratory, 2018) are shown as a reference in black. See Extended Data Figure 1-1 for growth-corrected and individual weight curves.

Twelve mice (five females and seven males, C57BL/6, The Jackson Laboratory, 6–17 months) were co-housed with their siblings. One animal was overweight at the start of the experiment (>45 g, body condition score 5), and was excluded; we thus show the data for 11 mice in Figure 1*D*. No animals showed signs of dehydration, or were dropped from the study. Each animal’s baseline weight was recorded on five consecutive days, while they had free access to plain water in their home-cage. We then switched their home-cage water to 2% CA, and recorded their daily weight. Laboratory shutdowns because of COVID-19 required us to cease this experiment, preventing a longer-term assessment of the effects of 2% CA water on body weight in older mice.

Mice in this study were on average 44 weeks old on the first day of the experimental intervention. Expected weight curves were computed from weighings of age-matched animals not undergoing fluid restriction, from The Jackson Laboratory (2018).

### Cohort 3

**Figure 2.**
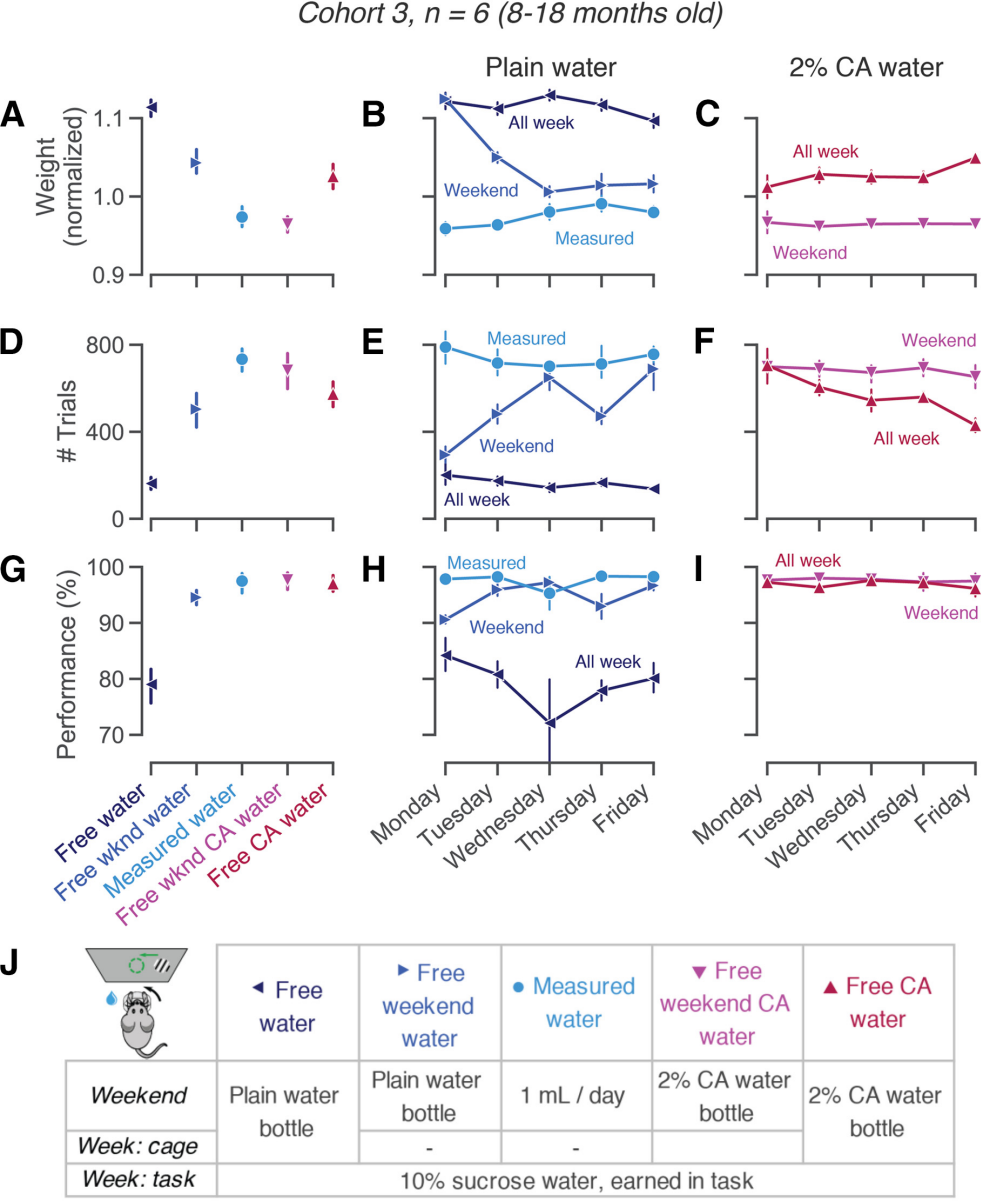
Mice perform many trials, even with free access to CA water. ***A***, Mouse weights per condition, normalized to each animal’s average weight over the course of the experiment. ***B***, ***C***, Weights as in ***A***, per weekday. ***D***, Trials performed per condition. ***E***, ***F***, Trials as in ***D***, per weekday. ***G***, Performance on easy trials (50% or 100% visual contrast), per condition. ***H***, ***I***, Performance as in ***G***, per weekday. Error bars show mean ± 68% CI across animals. ***J***, Schematic of all liquid regimes. Left top corner shows a schematic of the behavioral task. See Extended Data Figure 2-1 for day-by-day data throughout the experiment and Extended Data Figure 2-2 for comparisons with CA dissolved in HydroGel.

Six mice (male C57BL/6, Charles River, 8–18 months) were single-housed. On each weekday, animals performed a decision-making task (full task, as described below). We then varied the liquid regimes, with different types of water administration both during the weekend and weekdays (Fig. 2*J*). Home-cage bottles were put into the cage on Friday evening and removed on Monday morning, except for the conditions where free water (regular or 2% CA) was also available on weekdays. When no home-cage bottle was available and mice did not earn their minimum required amount of 1 ml/d, they were supplemented with measured water or HydroGel at the end of the day. No animals showed signs of dehydration, or were dropped from the study.

10.1523/ENEURO.0230-20.2020.f1-1 Extended Data Figure 1-1 Growth-corrected weight curves. ***A***, Data as in Figure 1*A*, but for growth-corrected weight curves. These were computed by expressing each animal’s baseline-corrected weight as a fraction of a sex-matched expected growth curve (data from The Jackson Laboratory, 2015). ***B***, As in ***A***, but each animal shown individually. Young animals on CA water reached stable, growth-corrected weights of 78–95% (averaged over days 20–30). ***C***, Data as in Figure 1*D*, for each animal shown individually. Adult animals on CA water reached stable weights of 78–91% (averaged over days 7–14). Download Figure 1-1, TIF file.

10.1523/ENEURO.0230-20.2020.f2-1 Extended Data Figure 2-1 Weight, trial counts and behavioral performance over time. Data as in Figure 2, shown over the full period of data collection. A leak in the rig tubing resulted in inaccurate reward volumes during two weeks of training (in late June and early September); these data were excluded from all analyses. Download Figure 2-1, TIF file.

10.1523/ENEURO.0230-20.2020.f2-2 Extended Data Figure 2-2 CA can be dissolved in HydroGel instead of water. If bottled CA water cannot easily be provided (during travel, due to cage size restrictions, or when head implants preclude the use of bottle-top cages), CA can be mixed into HydroGel cups (https://www.clearh2o.com/product/hydrogel/) as an alternative to liquid water. HydroGel was melted by placing unopened 56-g cups in a 60°C oven until the gel had liquified (3–5 h). CA powder was then mixed into the liquefied gel and stirred thoroughly, before resealing the HydroGel cups and letting them solidify at 4°C. As the flavor and perceived aversiveness of CA may differ when dissolved in water or HydroGel, we again titrated CA concentrations to achieve stable animal weights. The observation that higher concentrations of CA are required in HydroGel to achieve the same behavioral effects agreed with informal human flavor perception of both substances. ***A***, In a first cohort of animals (cohort 5: *n* = 5, 15 months), this required increasing concentrations from 1% to 6% m/v. Weight (from baseline on daily measured water), as animals were given free HydroGel with different concentrations of CA. ***B***, With a second cohort of animals (cohort 6: *n* = 10, 15–16 months), switching from plain HydroGel to 6% CA HydroGel resulted in weights close to the institutional minimum of 80%. At 5% CA HydroGel, all animals showed stable weights. ***C***, In our cohort of trained animals (Fig. 2), we confirmed that a weekend regime of 2% CA could be replaced by 5% CA in HydroGel. As in Figure 2, but comparing 2% CA water with 2% CA HydroGel and 5% CA HydroGel on weekends (same animals as shown in Fig. 2; see also Extended Data Fig. 2-1). Neither weekly trial counts (*t*_(5)_ = –0.932, *p* = 0.3941, Bf_10_ = 0.522) nor performance on easy trials (*t*_(5)_ = –1.444, *p* = 0.2083, Bf_10_ = 0.771) were significantly different in weeks following 2% CA water versus 5% CA HydroGel. Download Figure 2-2, TIF file.

### Cohort 4

We re-analyzed data of mice that completed training on a visual decision-making task (both basic and full tasks, see below) before 23 March 2020. All animals (140 male and female C57BL/6J, experiments performed across seven institutions) started with a week of water restriction, handling and habituation, after which they began training. On weekdays, if they did not earn all their required water in the task, they were supplemented with HydroGel. On weekends, mice received either measured water or HydroGel (40 μl/g of body weight or 1 ml/d, depending on institutional protocols), or free access to 2% CA water. See The International Brain Laboratory et al., 2020) for detailed descriptions of the apparatus, handling and husbandry, and automated training protocols. Data are available at https://data.internationalbrainlab.org.

### Decision-making task

Mice learned to perform a visual decision-making task where they detected the presence of a visual grating to their left or right, and reported the perceived location by turning a steering wheel (The International Brain Laboratory et al., 2020). The stimulus moved with the wheel rotation, remaining on the screen until feedback. For each correct response, mice received 1.5 μl of 10% sucrose water. Trial duration was estimated from stimulus onset to the delivery of feedback (either the water reward or a white noise indicating an error). Visual stimuli were presented at variable contrast (0–100%), allowing us to fit psychometric functions to quantify each animal’s behavior. The duration of each session was dependent on the engagement of the animal, which was determined through automated criteria (The International Brain Laboratory et al., 2020; see their Fig. 1*e* and Supplemental Table 3). Early in training, animals were supplemented toward the institutional minimum of 1 ml/d or 40 μl/g of body after the experimental session. After they became proficient at the behavioral task, they usually performed enough trials to fully earn their water requirement in the rig on weekdays.

A mouse was considered proficient at this basic task once its behavior met a set of prespecified criteria: >400 trials performed in each of the last three sessions; accuracy >80% correct on easy trials in each of the last three sessions; and on a psychometric function fit on those three sessions combined, a threshold of <19, absolute bias of <16, and lapses <0.2 (The International Brain Laboratory et al., 2020; Appendix 2). Extended Data Figure 3-1*E–I* shows behavioral data and psychometric function fits from the 3 d leading up to the animal being trained. The psychometric function has the following form:

$$\psi (x)=\gamma +(1-\gamma -\lambda )erf(\frac{x-\mu }{\sigma }+1)/2,$$

where x is the signed visual contrast, μ is a stimulus-independent bias term, σ is the steepness of the psychometric function, and γ and λ are lapse rates. After reaching task proficiency, animals proceeded to a more complex full task, where they combined visual information with an asymmetric stimulus prior that switched between blocks (The International Brain Laboratory et al., 2020).

### CA water preparation

CA (Sigma-Aldrich) was dissolved into tap water at 1% or 2% mass/volume (m/v). That is, at 2% m/v, 2 g of CA powder was mixed into 100-ml water and shaken until dissolved. Bottles were replaced with fresh CA water weekly, or when empty.

### Data analysis

Data about each animal (sex, date of birth, lineage), their water intake and weights were logged in the Alyx colony management system (The International Brain Laboratory et al., 2019) and analyzed using DataJoint (Yatsenko et al., 2018). We visualized all data in python, using pandas and seaborn (Waskom et al., 2020). Statistics were done using the Pingouin package (Vallat, 2018).

**Figure 3.**
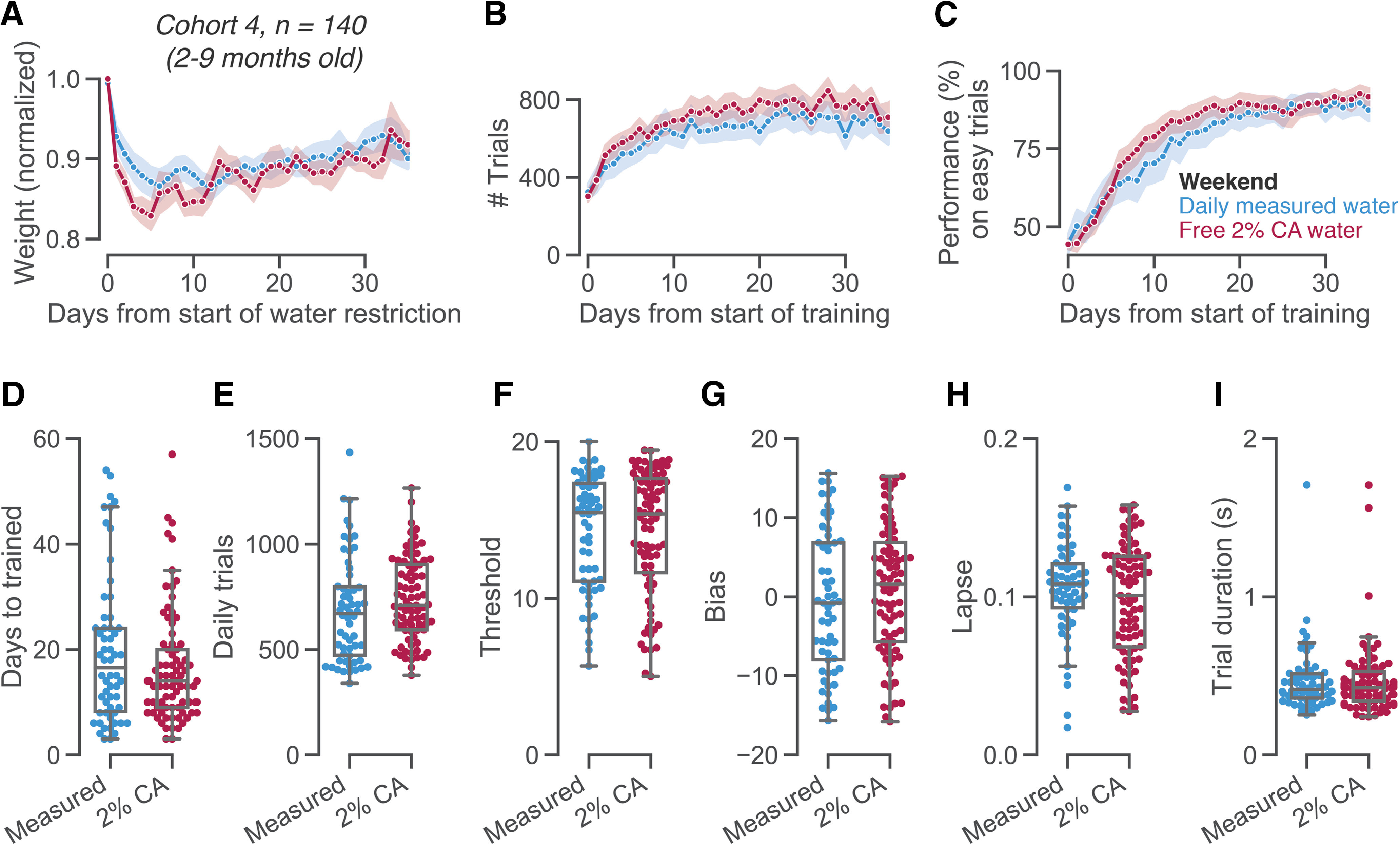
Weekend CA water does not adversely affect learning behavior. ***A***, Weight curves (normalized by each animal’s free water weight), separately for animals receiving measured water (in accordance with local IACUC protocols) or free 2% CA water on weekends and other non-training days. ***B***, Trial counts in a decision-making task over the course of learning. Training started ∼8 d after the beginning of water restriction. ***C***, Learning curves, showing performance on easy trials with 50% or 100% visual contrast. ***D****–****I***, Various measures of learning rates and stable behavior, separated by the weekend regime used in each lab. ***D***, Number of days until reaching training criteria. ***E****–****I***, For the three days over which animals passes training criteria, (***E***) average number of trials performed per day, (***F***) threshold from a psychometric function fit, (***G***) choice bias from a psychometric function fit, (***H***) lapse rate from a psychometric function fit, and (***I***) median trial duration. See Extended Data Figure 3-1 for an examination of sex differences.

For psychometric function parameters, as well as learning rates, daily trial counts and trial durations, we report statistics from an independent *t* test to test the effect of weekend water regime (Fig. 3). The degrees of freedom were corrected for unequal variances using a Welch–Satterthwaite correction. The corresponding Bayes factor indicates evidence for the null hypothesis of no difference when <1.

10.1523/ENEURO.0230-20.2020.f3-1 Extended Data Figure 3-1 Sex differences. We tested whether the weekend water regime (2% CA bottle vs measured water) differently affected (***A***) female and (***B***) male mice. Female mice given measured water on weekends learned the task slightly slower than female mice given 2% CA on weekends (*t*_(27)_ = –2.89, *p* = 0.007, Bf_10_ = 7.731). This was not the case for male mice (*t*_(77)_ = 0.30, *p* = 0.762, Bf_10_ = 0.242). Learning speeds showed a main effect of sex, and a significant interaction between water regime and sex (two-way ANOVA: effect of sex *F*_(1)_ = 14.367, *p* < 0.001; effect of water regime *F*_(1)_ = 4.645, *p* = 0.033; interaction *F*_(1)_ = 9.457, *p* = 0.003). The overall slower learning speeds of female mice may be due to their lower weights, causing them to be satiated more quickly and performing fewer trials early in the training process (we gave all animals a fixed reward volume, independent of their body weight). We can speculate that animals’ weight and hydration balance may be slightly different in different water regimes, which interacts with motivation and learning speed in a sex-specific manner. Learning speeds differ between labs, which may be caused by various factors (The International Brain Laboratory et al., 2020). Further work is thus needed disentangle any sex differences in the effects of water regime on task learning. There was no significant effect of sex, or interaction between sex and water regime, for stable behavior upon training completion: daily trial counts (effect of sex *F*_(1)_ = 0.345, *p* = 0.558; effect of water regime *F*_(1)_ = 2.070, *p* = 0.153; interaction *F*_(1)_ = 0.061, *p* = 0.806), visual threshold (effect of sex *F*_(1)_ = 0.142, *p* = 0.707; effect of water regime *F*_(1)_ = 0.002, *p* = 0.962; interaction *F*_(1)_ = 0.584, *p* = 0.446), choice bias (effect of sex *F*_(1)_ = 0.825, *p* = 0.365; effect of water regime *F*_(1)_ = 1.374, *p* = 0.243; interaction *F*_(1)_ = 0.027, *p* = 0.869), lapse rate (effect of sex *F*_(1)_ = 2.085, *p* = 0.151; effect of water regime *F*_(1)_ = 2.284, *p* = 0.133; interaction *F*_(1)_ = 0.320, *p* = 0.573), or trial duration (effect of sex *F*_(1)_ = 2.730, *p* = 0.101; effect of water regime *F*_(1)_ = 0.064, *p* = 0.801; interaction *F*_(1)_ = 2.457, *p* = 0.119). Download Figure 3-1, TIF file.

### Code and data availability

All code and instructions for reproducing figures and statistics are available at https://github.com/int-brain-lab/citricAcid.

## Results

### Mice maintain health weights and water intake on CA water

We first confirmed that mice remained healthy and maintained stable weights when given free access to CA water (Fig. 1*A*). Three subgroups of young animals (cohort 1: total *n* = 17, 2-3 months old) were given measured amounts of daily water, free plain water, or free CA water in their home-cage. All animals were healthy (as judged by frequent experimenter handling and inspection) and showed no signs of dehydration. Over time, mice with access to free plain water steadily gained weight (Fig. 1*A*, dark blue), as expected for young C57BL/6J mice with access to free food and water (Fig. 1*A*, thick black line). Mice on measured water rapidly lost weight over the first week, after which their weight loss reversed (Fig. 1*A*, light blue line), matching previous reports (Guo et al., 2014). Mice with access to CA water at a low concentration (1% m/v) did not show adverse health effects nor signs of dehydration, and lost only modest amounts of weight (Fig. 1*A*, red line). These animals also tolerated CA at a higher concentration of 2% m/v (Fig. 1*A*), in line with findings in rats (Reinagel, 2018). With free access to CA water, animals’ weights stabilized after about a week (Fig. 1*A*, days 11–30).

Since these young animals (on average nine weeks old at the start of the experiment) were still growing, their weights did not solely reflect the water regime. Taking into account this expected weight gain with age, animals on free CA water showed stable weights around 78–95% (Extended Data Fig. 1-1*A*,*B*). We also repeated weight measurements in older mice (Fig. 1*D*). After switching to free CA water, animals were healthy and maintained stable weights (Fig. 1*D*). In the second week after introducing 2% CA water, animals retained on average 83% (78–91%) of their baseline body weight (Extended Data Fig. 1-1*C*). While a weight loss of 20% is considerably larger than for rats with free access to 2% CA water (who retained 97% of their baseline body weight, Reinagel, 2018), it is similar to those stable weights obtained under widely used water restriction protocols (75–85% after several weeks of water restriction; Guo et al., 2014).

As expected from CA-induced weight loss, animals with access to CA water reduced their fluid intake (Fig. 1*B*,*C*). Mice consumed 3.9 ml of plain water per day (range 2.6–6.3 ml; Fig. 1*B*, blue), but only about one third as much 2% CA water (1.3 ml, range 1.3–1.5 ml; Fig. 1*B*, red). Importantly, mice with access to free 2% CA water were still motivated to drink plain water. In 5 min of free access, they drank 0.58–0.99 ml of plain water, similar to animals receiving daily measured water (Fig. 1*C*). Not only did animals tolerate 2% CA water well, these data also suggest that free access to CA water might not impede animals’ motivation to perform behavioral tasks to earn more palatable liquids.

### Mice perform many trials, even with free access to CA water

In a new cohort of animals (cohort 2: *n* = 6, 8–18 months) that had been trained on the visual decision-making task (The International Brain Laboratory et al., 2020), we tested how different liquid regimes affected animals’ willingness to work for sweetened water rewards (Fig. 2). Animals performed a decision-making task for 45–90 min each weekday and earned a drop of 10% sucrose water for each correct response. The length of each session was dictated by the automated detection of the animal’s engagement, allowing them to work to satiety (The International Brain Laboratory et al., 2020; see their Fig. 1*e* and Supplemental Table 3). Expert animals usually earned more than their minimum daily water requirement (determined by institutional protocols) in the task and did not require supplemental fluids. In this cohort, we then varied the liquid regime from week to week over several months (Fig. 2*J*; Extended Data Fig. 2-1).

On weekends and other non-training days, mice are commonly given either daily measured water, or free access to a bottle in their home-cage. When mice drank measured amounts (1 ml) of plain water on weekends (light blue circle), they performed sessions of 730–1230 trials stably over the week (Fig. 2*E*). This practice is commonly used in laboratories that perform high-throughput mouse training. It is also the most laborious for the experimenter, who needs to provide measured amounts of water each day of the weekend. On the contrary, free home-cage water during the weekend (blue, rightward-pointing triangle) resulted in lower trial counts and worse performance on Mondays and Tuesdays (97–420 trials on Mondays vs 668–1140 trials on Fridays; *t*_(5)_ = −3.951, *p* = 0.0109, Bf_10_ = 6.491; Fig. 2*E*,*H*; Busse et al., 2011). Under this regime, weights often fluctuated dramatically over the course of the week (weight loss of around 10% of body weight or ∼3 g between Mondays and Fridays; Fig. 2*B*), which may have adverse effects on animal health (Rowland, 2007).

Free access to 2% CA water over the weekend (pink, downward pointing triangle) combines the best of both approaches. Without requiring experimenter intervention on days without training, animals maintained stable weights (Fig. 2*C*), and consistently performed many trials (Fig. 2*F*). We could not detect a significant difference in the number of trials performed after a weekend with free 2% CA water (682 trials/d, range 535–826) versus measured water (733 trials/d, range 634–817; *t*_(5)_ = 0.880, *p* = 0.4193, Bf_10_ = 0.505; Fig. 2*D*). Similarly, high trial yields were achieved by providing CA dissolved in HydroGel cups, a practical alternative in cases where bottled water is difficult to provide (Extended Data Fig. 2-2).

Free CA water yields the most trials when given only on weekends, but can also be kept in the home-cage throughout the week. When free 2% CA was available continuously (red, upward pointing triangle), the number of trials slightly decreased from Monday to Friday (435–1019 on Mondays vs 312–513 on Fridays; *t*_(5)_ = 3.002, *p* = 0.0300, Bf_10_ = 3.035; Fig. 2*F*). As a result, the average number of trials was slightly lower than when the 2% CA water bottle was removed on Monday morning (average 573 vs 682 trials, *t*_(5)_ = −2.505, *p* = 0.0542, Bf_10_ = 1.971; Fig. 2*D*). While both continuous and weekend-only CA water allow for stable health and fairly high motivation, a regime with continuous 2% CA water availability may not consistently yield the high trial numbers required for some experimental purposes.

### Weekend CA water does not adversely affect learning behavior

Since mice maintained high motivation with free CA water on weekends, we implemented this strategy across several laboratories in a large-scale neuroscience collaboration (The International Brain Laboratory et al., 2020). All animals underwent standardized surgeries and training protocols to learn a visual decision-making task (The International Brain Laboratory et al., 2020). Because of differences in local animal care arrangements and licenses, two different protocols were used on weekends: four labs (82 mice) used free 2% CA water, whereas the other five labs (58 mice) gave measured water or HydroGel. This allowed us to investigate the effects of CA water in a large cohort of animals, trained in different laboratories across seven institutions.

Across all labs, and regardless of the weekend water regime, animals successfully learned the task over the course of a few weeks (Fig. 3*B*,*C*). Both water regimes also resulted in similar weight curves, with a characteristic rapid drop and subsequent slow weight gain as animals learned to earn sucrose water in the task (Fig. 3*A*). The number of days needed to complete training (determined as reaching a specified set of behavioral criteria; The International Brain Laboratory et al., 2020; see their Supplemental Table 2), did not vary with weekend water strategy (*t*_(101)_ = −1.47, *p* = 0.145, Bf_10_ = 0.491; Fig. 3*D*). Upon training completion, multiple measures of animal behavior were indistinguishable between those labs using measured water versus CA water on weekends: the number of trials performed per day (*t*_(104)_ = 1.33, *p* = 0.188, Bf_10_ = 0.410; Fig. 3*E*), visual threshold (*t*_(128)_ = 0.10, *p* = 0.919, Bf_10_ = 0.185; Fig. 3*F*), choice bias (*t*_(116)_ = 1.05, *p* = 0.297, Bf_10_ = 0.304; Fig. 3*G*), lapse rate (*t*_(133)_ = −1.36, *p* = 0.175, Bf_10_ = 0.429; Fig. 3*H*), and median trial duration (*t*_(128)_ = −0.03, *p* = 0.978, Bf_10_ = 0.184; Fig. 3*I*). These patterns were similar for both male and female mice (Extended Data Fig. 3-1). This suggests that CA water is a reliable alternative to water restriction for achieving high quality mouse behavior.

## Discussion

High-throughput, reliable behavioral training is a key requirement for the use of mice in behavioral and systems neuroscience. Here, we have shown that free access to CA water is well tolerated, and motivates mice to perform many trials of a decision-making task in which they earn sugar water. We thus consider CA water a promising alternative to water restriction for some experimental regimes.

In contrast to commonly used water restriction regimes, free access to CA water allows animals full control over the amount and timing of their water intake. Home-cage access to food and CA water also enables animals to eat and drink simultaneously, which benefits their metabolism and nutrition (Fertig and Edmonds, 1969; Toth and Gardiner, 2000). In some institutions, drinking water is acidified to reduce bacterial growth (Reinagel, 2018). Since 2% CA water has pH 2.07, it may convey antibacterial properties that benefit animal welfare.

Beyond extending previous experiments with CA water from rats (Watson et al., 1986; Reinagel, 2018) to mice, we here propose a new variant that maintains high trial yields. Young rats with continuous access to 2% CA water perform only around 68% of their water-restricted trial counts during daily sessions (84% for live-in behavioral testing; Reinagel, 2018). Similarly, when CA water was available throughout the week, mice performed around 22% fewer trials compared with usual conditions (no home-cage water available on testing days, measured water on weekends). Such continuous CA accessibility may thus be suitable if high trial yields are not needed, or if the baseline trial count is sufficiently high that an acceptable number of trials can be collected. As an alternative, we here propose that providing CA water only on weekends and other non-training days balances animal welfare with ease of use and behavioral throughput.

While we have achieved a satisfactory balance between water intake and trial counts using a concentration of 2% CA (m/v), this concentration could be further fine-tuned based on the details of each experimental setup. Trial yield may also depend on individual animal’s taste perception (which likely varies between species and strains), task design (e.g., head-fixed vs freely moving), and local factors such as food or environmental humidity. For instance, if only CA water is available in the home-cage, sweetened task water likely further increases animals’ willingness to work. The strategy of placing animals on free CA water over the weekend may not be viable for all experiments: for example, studies investigating differences in reward and taste processing, or those requiring precise tracking of individual animal’s fluid intake.

Beyond benefits to animal welfare, eliminating water restriction also benefits the quality and throughput of behavioral experiments. It removes the need to give animals supplemental fluids on days when they do not train (e.g., weekends) or days when they do not earn their water requirements in the task (as often happens early in training). Such supplements are often beyond the capacities of routine animal care procedures, and require scientific personnel’s daily attention and record-keeping. Besides being highly labor-intensive, water supplements carry a risk of human error in the timing and precise delivery of required water amounts, as well as the distribution of water between co-housed mice. Unexpected absence of experimental personnel usually requires animals to be placed on free plain water, which can impede their progress in behavioral training for many days or weeks afterward.

Free access to CA water provides a relatively non-labor-intensive, low-error option for keeping animals healthy without hindering behavioral training progress. This suggests that CA water can serve as a reliable and standardized strategy to achieve high quality task behavior, further facilitating the use of mice in high-throughput behavioral studies.

10.1523/ENEURO.0230-20.2020.ed1 Extended Data Ed1 Contributions table. Download Extended Data Ed1, TIF file.

## Synthesis

Reviewing Editor: Laura Bradfield, University of Technology Sydney Decisions are customarily a result of the Reviewing Editor and the peer reviewers coming together and discussing their recommendations until a consensus is reached. When revisions are invited, a fact-based synthesis statement explaining their decision and outlining what is needed to prepare a revision will be listed below. The following reviewer(s) agreed to reveal their identity: Shauna Parkes, Pamela Reinagel.

Dear Dr. Urai.

Thank you for submitting your manuscript “Citric acid water as an alternative to water restriction for high-yield mouse behavior” to eNeuro. It has now been evaluated by two expert reviewers and myself. We agree that the manuscript contains important work of considerable merit, but that there are several issues that need to be addressed before it is suitable for publication. These are listed below.

1. As previously discussed with the corresponding author, some of the sample sizes are relatively small. It was mentioned that a reason for this was due to lab shutdowns in compliance with COVID19 policies. Could the authors please make mention of this in the manuscript?

2. A ‘visual decision-making task’ is referred to several times and data for such a task included in the figures, yet I failed to find any description of the procedures for this task. Could the authors please detail the behavioural procedures of this task, possibly including a diagrammatic representation of it in one of the figures.

3. The manuscript needs to be re-organised in line with the requirements of eNeuro. For instance:

o The text should be double-spaced.

o The manuscript should include the following sections in the orders listed:

∘ Abstract (250 word maximum)

∘ Significance Statement (120 words maximum)

∘ Introduction

∘ Materials & Methods

∘ Results

∘ Discussion

∘ References

∘ Legends

∘ Multimedia, Figure, and Table

4. Please also respond to each individual points raised by each reviewer in the appended reviews below. In particular:

o Please ensure Extended data for Figure 1 is moved to the main text.

o Please acknowledge that 20% weight loss is not minor but is rather substantial.

o Please revise the statement that water restriction is less detrimental to the animal’s wellbeing than food restriction, as we did not find this statement to be well supported by the evidence.

Reviewers’ comments:

Reviewer #1

This manuscript nicely builds upon work by Pamela Reinagel exploring freely available citric acid water as an alternative to water restriction in rats. Here, the authors show that CA water is also a viable alternative to water restriction in studies using mice and, moreover, demonstrate that free CA on weekends does not result in any significant performance deficits. Overall, the study will be appreciated by a broad audience and will potentially lead to changes in how water restriction is managed in mice. I have only minor comments.

1. It wasn’t clear to me whether all of the data or only part of the data was collected as part of the International Brain Laboratory effort (available on bioRxiv). Could the authors please include a clearer statement regarding this? For instance, it was my understanding that the data reported in Figure 2 and 3 were collected as part of this international collaboration but not the data reported in Figure 1. Is that correct?

2. The statistics are reported in the main text for Figures 1 and 2 but not Figure 3 (stats are reported in the figure itself). Please use the same reporting for all data.

3. Data from male and female mice were used in Figure 3. Is it possible for the authors to comment on whether or not any differences were observed between males and females in the use of measured versus free 2% CA water on weekends? And, if possible, provide the appropriate analyses. This could be extremely useful.

4. The authors report three sets of results as part of the extended data, all of which are integral to the study and the correct intepretation of the results. However, the authors make some claims regarding the extended data that don’t appear to be supported by any statistics. For example, in reference to the hydrogel data, the authors state “In our cohort of trained animals (Figure 2), we confirmed that weekend regimes of 2% CA in water versus 5% CA in Hydrogel yielded similar trial counts and task performance (Extended Data, Figure 2 - 1 and Figure 2 - 2)” but I cannot find the statistics to support this statement. Also, it would be useful if the authors explained the colour coding for Figure 2-1 in the legend.

Reviewer #2

This manuscript generalizes to mice an approach recently demonstrated in rats for training with water rewards without water restriction. This required establishing two main claims: that a weak citric acid solution is a safe and sufficient water source for mice, and that mice with ad lib citric acid solution still perform trials for water rewards. The data provided here validate this method for mice in short daily training sessions. It shows that giving free CA water continuously has positive health benefits with a modest reduction in trial rate, as shown before for rats trained in short daily sessions. Moreover giving free CA instead of measured water on non-training-days (weekends) had no down-sides for animal health or task performance, while being far less costly/labor intensive. This result will be of practical value to a very large number of neuroscience labs.

Major points

In the abstract, “and propose a variant of the method that preserves high trial yield....” is unnecessarily vague, why not state what specific changes preserved high trial yield relative to the previous method? At first I assumed you meant using sweetened water, but this was never varied or discussed, so now I assume you mean the variant of giving CA water only on weekends, which changed the 22% drop in trial rate you saw on 7-day ad-lib CA to a nonsignificant change in trial rate with weekend CA water?

In the abstract, “Free access to 2% CA water resulted in stable weights” should be changed to: “In adults, free access...” unless you can show stabilization of growth-curve-corrected weights for the juveniles (see point 5 below).

Same sentence “weights stabilized at 80% of baseline”. A 20% weight loss is substantial enough to be worth mentioning. Any speculation why you observed so much more weight loss in adult mice than previously observed in adult rats (maintained 97% of baseline in Reinagel 2018)?

Clarify claims in introduction: I think you mean to claim: “Mice with free access to CA water on weekends were motivated to perform many trials (500-1000 per day) evenly over the week”. As currently worded, it was unclear if the claim was about the weekend vs. the all-week CA protocol, or if the trial numbers were per day or per week.

Figure 1a: in juveniles weight loss should be computed relative to the growth-curve projected weight, not relative to the initial weight. Showing these curves is fine, normalizing each animal’s growth curve to the rat’s initial weight is fine. But describing this result as “stable weight” is not justified by this graph. Please state the maximum weight loss at any time point and the stabilized weight loss as percentage of the growth-curve-corrected expected weight within animal. If growth-adjusted weight stabilizes at >80% the current claims are fine, otherwise claims should be modified to reflect this.

Please comment on the criteria for temporary or permanent removal of an animal from water restriction (e.g. maximum percent weight loss?) and state whether any animals needed to be temporarily or permanently taken off water restriction due to these criteria, or any dropped from the study for inadequate trial numbers, or any incidence of mortality due to accidental dehydration. If any of these events occurred, please comment on whether the rates of such events differed on the weekend-CA or 7-day CA protocol to the measured water protocol.

Minor points

In several places you state that rats performed fewer or ∼30% fewer trials than under water restriction, which was true when CA was 2% and access to the task was limited to 2 hours/day. This is an appropriate comparison to your training protocol for mice, but should be specified. (In Reinagel 2018 in the live-in task, trial rates were 84% of restricted condition with 2%CA, and indistinguishable from water restriction with 4%CA).

"As opposed to food restriction, water restriction only mildly affects animals’ wellbeing” This statement surprised me. Caloric restriction increases lifespan and many other health metrics, and as such can be considered beneficial, whereas water restriction has no known benefits. Many labs’ reasons to avoid food restriction is rather that consumption of reward pellets takes a lot of time and creates chewing artifacts in electrophyisiological recordings or motion artifacts in imaging.

Discussion says you saw a 16% drop in trial rates on ad lib CA compared to “usual conditions” but isn’t it 22% (573 vs 733?). The 16% drop was between weekend CA and ad-lib CA (573 vs 682).

Using sweetened reward was another difference between this study and the original rat paper. Might this account for why you obtained only 22% drop in trial rate on your 7-day-free-CA protocol compared to restriction, vs. the 34% drop in the comparable experiment in rat?

## Author Response

Dear Editor and Reviewers,

Thank you for your positive assessment of our work, and your constructive comments and suggestions. We have now revised the manuscript, which we believe has been substantially strengthened by these changes. Please see in-line answers to each of your points below.

On behalf of all authors,

Anne Urai

Thank you for submitting your manuscript “Citric acid water as an alternative to water restriction for high-yield mouse behavior” to eNeuro. It has now been evaluated by two expert reviewers and myself. We agree that the manuscript contains important work of considerable merit, but that there are several issues that need to be addressed before it is suitable for publication. These are listed below.

1. As previously discussed with the corresponding author, some of the sample sizes are relatively small. It was mentioned that a reason for this was due to lab shutdowns in compliance with COVID19 policies, and that open data was later used with a larger sample size to replicate earlier findings, reducing concerns about reproducibility. Could the authors please make mention of this in the manuscript?

We agree and have now made this point explicit in the Methods section: ‘Laboratory shutdowns due to COVID-19 required us to cease this experiment, preventing a longer-term assessment of the effects of 2% CA water on body weight in older mice.’

2. A ‘visual decision-making task’ is referred to several times and data for such a task included in the figures, yet I failed to find any description of the procedures for this task. Could the authors please detail the behavioural procedures of this task, possibly including a diagrammatic representation of it in one of the figures.

We have now added a Methods section ‘Decision-making task’ with a short summary. We refer the reader more explicitly to International Brain Laboratory et al. 2020 (https://doi.org/10.1101/2020.01.17.909838) for a further, more detailed description of all task parameters, automated training regime stages, and hardware and software implementations of the task. We also added a task schematic to Figure 2j.

3. The manuscript needs to be re-organised in line with the requirements of eNeuro.

The manuscript organization is now in line with eNeuro’s formatting requirements. For completeness, we have also added the institutional animal ethics information and the author names and affiliations back into the manuscript.

4. Please also respond to each individual points raised by each reviewer in the appended reviews below, with particular attention to:

∘ Please ensure Extended data for Figure 1 is moved to the main text.

∘ Please acknowledge that 20% weight loss is not minor but is rather substantial.

∘ Please revise the statement that water restriction is less detrimental to the animal’s wellbeing than food restriction, as we did not find this statement to be well supported by the evidence.

All three suggestions have been incorporated. Please see below for more in-depth responses to each of the reviewers’ comments.

Reviewers’ comments:

Reviewer #1

This manuscript nicely builds upon work by Pamela Reinagel exploring freely available citric acid water as an alternative to water restriction in rats. Here, the authors show that CA water is also a viable alternative to water restriction in studies using mice and, moreover, demonstrate that free CA on weekends does not result in any significant performance deficits. Overall, the study will be appreciated by a broad audience and will potentially lead to changes in how water restriction is managed in mice. I have only minor comments.

Thank you for your positive assessment of our work, and your helpful comments.

1. It wasn’t clear to me whether all of the data or only part of the data was collected as part of the International Brain Laboratory effort (available on bioRxiv). Could the authors please include a clearer statement regarding this? For instance, it was my understanding that the data reported in Figure 2 and 3 were collected as part of this international collaboration but not the data reported in Figure 1. Is that correct?

Indeed, the data was collected under the umbrella of the IBL consortium - but only partially overlaps with the large dataset described on bioRxiv. This is now clarified in the Methods section, under ‘Experimental Design’: “The data shown in Figure 3 (cohort 4) are part of a large, public dataset (described in (International Brain Laboratory et al., 2020) and available at https://data.internationalbrainlab.org). All other experiments were collected separately (although in the case of cohort 3, using the same behavioral task and apparatus as described in (International Brain Laboratory et al., 2020)).”

2. The statistics are reported in the main text for Figures 1 and 2 but not Figure 3 (stats are reported in the figure itself). Please use the same reporting for all data.

The statistics are now reported in-text throughout.

3. Data from male and female mice were used in Figure 3. Is it possible for the authors to comment on whether or not any differences were observed between males and females in the use of measured versus free 2% CA water on weekends? And, if possible, provide the appropriate analyses. This could be extremely useful.

Thank you for this suggestion. We now include an Extended Data, Figure 3-1 where we split all analyses in Figure 3 by mouse sex. We only observed one differential effect of CA water for males vs. females: females on weekend CA water learned the task quicker than those on restricted water. After becoming proficient at the task, males and females did not differ in their behavior depending on the weekend water regime. We further discuss this finding in the text corresponding to Extended Data, Figure 3-1.

4. The authors report three sets of results as part of the extended data, all of which are integral to the study and the correct interpretation of the results. However, the authors make some claims regarding the extended data that don’t appear to be supported by any statistics. For example, in reference to the hydrogel data, the authors state “In our cohort of trained animals (Figure 2), we confirmed that weekend regimes of 2% CA in water versus 5% CA in Hydrogel yielded similar trial counts and task performance (Extended Data, Figure 2 - 1 and Figure 2 - 2)” but I cannot find the statistics to support this statement. Also, it would be useful if the authors explained the colour coding for Figure 2-1 in the legend.

Thank you for noticing this. We have now added appropriate statistics into the text that goes with Figure 2-2: “Specifically, neither weekly trial counts (t(5) = -0.932, p = 0.3941, Bf10 = 0.522) nor performance on easy trials (t(5) = -1.444, p = 0.2083, Bf10 = 0.771) were significantly different in weeks following 2% CA water vs. 5% CA HydroGel.” We also added a legend to Figure 2-1.

Reviewer #2

This manuscript generalizes to mice an approach recently demonstrated in rats for training with water rewards without water restriction. This required establishing two main claims: that a weak citric acid solution is a safe and sufficient water source for mice, and that mice with ad lib citric acid solution still perform trials for water rewards. The data provided here validate this method for mice in short daily training sessions. It shows that giving free CA water continuously has positive health benefits with a modest reduction in trial rate, as shown before for rats trained in short daily sessions. Moreover giving free CA instead of measured water on non-training-days (weekends) had no down-sides for animal health or task performance, while being far less costly/labor intensive. This result will be of practical value to a very large number of neuroscience labs.

Major points

In the abstract, “and propose a variant of the method that preserves high trial yield....” is unnecessarily vague, why not state what specific changes preserved high trial yield relative to the previous method? At first I assumed you meant using sweetened water, but this was never varied or discussed, so now I assume you mean the variant of giving CA water only on weekends, which changed the 22% drop in trial rate you saw on 7-day ad-lib CA to a nonsignificant change in trial rate with weekend CA water?

Thank you for pointing out this unclear phrasing. We indeed intended to refer to the strategy of only providing free CA water on weekends, versus the whole week. The sucrose reward water is mostly used to boost trial counts overall; we did not test how the reward water interacts with home-cage CA water on weekends or during the week.

To avoid confusion, we have removed the phrase “and propose a variant of the method...” from the abstract. In the last paragraph of the abstract, we write “When free CA water was used instead of water restriction only on weekends, learning and decision-making behavior were unaffected.”

In the abstract, “Free access to 2% CA water resulted in stable weights” should be changed to: “In adults, free access...” unless you can show stabilization of growth-curve-corrected weights for the juveniles (see point 5 below).

We have added the clarification “In adult mice, ...” to this sentence in the introduction.

Same sentence “weights stabilized at 80% of baseline”. A 20% weight loss is substantial enough to be worth mentioning. Any speculation why you observed so much more weight loss in adult mice than previously observed in adult rats (maintained 97% of baseline in Reinagel 2018)?

Our mice indeed lost considerably more weight under CA water than the rats tested in Reinagel 2018, which we now acknowledge explicitly. However, this weight loss was comparable to water restriction protocols that are widely used in behaving mice, reported here and in the literature (Guo et al. 2014).

In the Results section of Figure 1d, we now briefly address this difference between rats and mice: “While a weight loss of 20% is considerably more severe than for rats with free access to 2% CA water (who retained 97% of their baseline body weight, Reinagel, 2018), it is similar to those stable weights obtained under widely used water restriction protocols (75-85% after several weeks of water restriction, (Guo et al., 2014)).”

Clarify claims in introduction: I think you mean to claim: “Mice with free access to CA water on weekends were motivated to perform many trials (500-1000 per day) evenly over the week”. As currently worded, it was unclear if the claim was about the weekend vs. the all-week CA protocol, or if the trial numbers were per day or per week.

We now rephrased this sentence, and switched its order with the sentence before: “Giving mice free access to CA water on weekends did not affect trial numbers nor task performance on subsequent training days. Specifically, mice with free access to CA water on weekends were motivated to perform many trials (500-1000 daily) evenly over the week, doing a decision-making task in which they earned sugar water.”

Figure 1a: in juveniles weight loss should be computed relative to the growth-curve projected weight, not relative to the initial weight. Showing these curves is fine, normalizing each animal’s growth curve to the rat’s initial weight is fine. But describing this result as “stable weight” is not justified by this graph. Please state the maximum weight loss at any time point and the stabilized weight loss as percentage of the growth-curve-corrected expected weight within animal. If growth-adjusted weight stabilizes at >80% the current claims are fine, otherwise claims should be modified to reflect this.

We now compute each animal’s growth-corrected weight by expressing their baseline-corrected weight changes (as in Figure 1a) as a fraction of an age- and sex-matched growth curve, taken from Jackson Laboratory. These growth-corrected curves, individual weight trajectories and weight ranges are now shown in Extended Data, Figure 1 - 1 and referred to in the main text.

Over the course of the experiment, the lowest growth-corrected weights were 77-92% across animals. Young animals reached stable, growth-corrected weights of 78-95%; these numbers were highly similar to the stable weights measured in adult animals.

The new paragraph in the Results section now reads:

"Since these young animals (on average 9 weeks old at the start of the experiment) were still growing, their weights did not solely reflect the water regime. Taking into account this expected weight gain with age, animals on free CA water showed stable weights around 78-95% (Extended Data, Figure 1 - 1a,b). We also repeated weight measurements in older mice (Figure 1d). After switching to free CA water, animals were healthy and maintained stable weights (Figure 1d). In the second week after introducing 2% CA water, animals retained on average 83% (78-91%) of their baseline body weight (Extended Data, Figure 1 - 1c). While a weight loss of 20% is considerably larger than for rats with free access to 2% CA water (who retained 97% of their baseline body weight, Reinagel, 2018), it is similar to those stable weights obtained under widely used water restriction protocols (75-85% after several weeks of water restriction, (Guo et al., 2014)).”

Please comment on the criteria for temporary or permanent removal of an animal from water restriction (e.g. maximum percent weight loss?) and state whether any animals needed to be temporarily or permanently taken off water restriction due to these criteria, or any dropped from the study for inadequate trial numbers, or any incidence of mortality due to accidental dehydration. If any of these events occurred, please comment on whether the rates of such events differed on the weekend-CA or 7-day CA protocol to the measured water protocol.

Thank you for pointing out this omission. We have now included an explicit statement about any animals dropping out, in each cohort’s description (see Methods).

In cohort 1 (CCU), 2, 3, 5, 6 (CSHL), animals would be removed from the study if they consistently weighed less than 80% of their free-water weight following three weeks after the start of the experiment. We occasionally supplemented animals with high-caloric foods to gain weight. Euthanasia would be performed if animals showed: weight loss below 75% of free weight, poor body condition, lethargy, loss of appetite, poor posture/appearance, or moderate to severe recurring dehydration and associated conditions. None of these events occurred. We also never observed spontaneous mortality due to dehydration.

In cohort 2, one animal dropped weight for two subsequent days, but subsequently recovered (Figure 1 - Supplement 1). Our best guess is that these data points reflect measurement error, as they were taken on a weekend by a different experimenter than usual. In cohort 6, we reduced the concentration of CA in HydroGel when some animals reached weights around 80% of their free-water weight, upon which all recovered.

In cohort 4 (IBL), we used the following guideline: when animals dropped below 85% or 80% (depending on institutional protocol) of their free-water weight, we first supplemented them with dried fruits, nuts and yoghurt drops. If this additional high-calorie food did not increase their weight, we temporarily gave animals free access to regular tap water until their weight reached >87%. Institutional protocols varied, and we did not track such recovery procedures or dehydration problems in a standardized way across the consortium. Therefore, we can unfortunately not offer a detailed analysis between labs.

Low trial numbers were mostly observed early in training; after learning the tasks, animals usually earned all their water in the task. If they failed to earn their minimum requirements, they were supplemented with HydroGel or water at the end of the day.

Minor points

In several places you state that rats performed fewer or ∼30% fewer trials than under water restriction, which was true when CA was 2% and access to the task was limited to 2 hours/day. This is an appropriate comparison to your training protocol for mice, but should be specified. (In Reinagel 2018 in the live-in task, trial rates were 84% of restricted conditions with 2% CA, and indistinguishable from water restriction with 4% CA).

We have clarified this in the Introduction:

"However, free access to 2% CA water led to a reduction in trial yield of around 30% in 2-hour daily training sessions (Reinagel, 2018), impeding its use in high-throughput behavioral paradigms. Moreover, it is not known if this approach would also work in mice.”

And in the Discussion:

"Young rats with continuous access to 2% CA water perform only around 68% of their water-restricted trial counts during daily sessions (84% for live-in behavioral testing; Reinagel, 2018).”

"As opposed to food restriction, water restriction only mildly affects animals’ wellbeing” This statement surprised me. Caloric restriction increases lifespan and many other health metrics, and as such can be considered beneficial, whereas water restriction has no known benefits. Many labs’ reasons to avoid food restriction is rather that consumption of reward pellets takes a lot of time and creates chewing artifacts in electrophysiological recordings or motion artifacts in imaging.

Thank you for pointing this out. We have removed this statement “As opposed to food restriction, water restriction only mildly affects animals’ wellbeing, and results in more robust performance on a variety of tasks (Tucci et al., 2006)” from the introduction.

Discussion says you saw a 16% drop in trial rates on ad lib CA compared to “usual conditions” but isn’t it 22% (573 vs 733?). The 16% drop was between weekend CA and ad-lib CA (573 vs 682).

Thank you for noticing this, we have now corrected this and clarified the sentence:

"Similarly, when CA water was available throughout the week, mice performed around 22% fewer trials compared to usual conditions (no home-cage water available on testing days, measured water on weekends).”

Using sweetened reward was another difference between this study and the original rat paper. Might this account for why you obtained only a 22% drop in trial rate on your 7-day-free-CA protocol compared to restriction, vs. the 34% drop in the comparable experiment in rats?

We have now addressed this in the Discussion: “Trial yield may also depend on individual animal’s taste perception (which likely varies between species and strains), task design (e.g. head-fixed vs. freely moving), and local factors such as food or environmental humidity. For instance, if only CA water is available in the home cage, sweetened task water likely further increases animals’ willingness to work.”
