# Supplementary figures and images for: Citric Acid Water as an Alternative to Water Restriction for High-Yield Mouse Behavior
Source: eNeuro. 2021 Feb 2;8(1):ENEURO.0230-20.2020. doi: 10.1523/ENEURO.0230-20.2020 (PMC7890523; doi:10.1523/ENEURO.0230-20.2020)

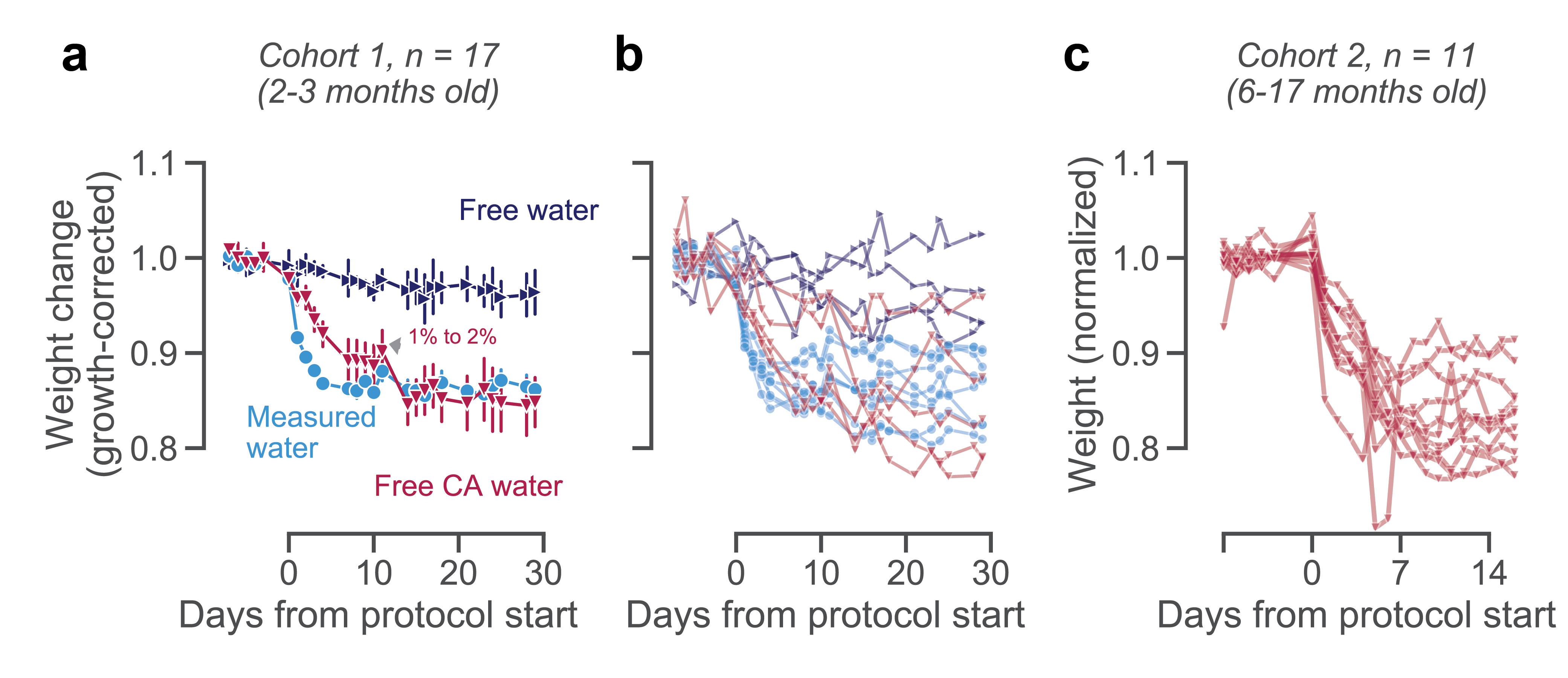

Supplement: Extended Data Figure 1-1 — Growth-corrected weight curves. A, Data as in Figure 1A, but for growth-corrected weight curves. These were computed by expressing each animal’s baseline-corrected weight as a fraction of a sex-matched expected growth curve (data from The Jackson Laboratory, 2015). B, As in A, but each animal shown individually. Young animals on CA water reached stable, growth-corrected weights of 78–95% (averaged over days 20–30). C, Data as in Figure 1D, for each animal shown individually. Adult animals on CA water reached stable weights of 78–91% (averaged over days 7–14). Download Figure 1-1, TIF file. [file enu-eN-MNT-0230-20-s01.tif]

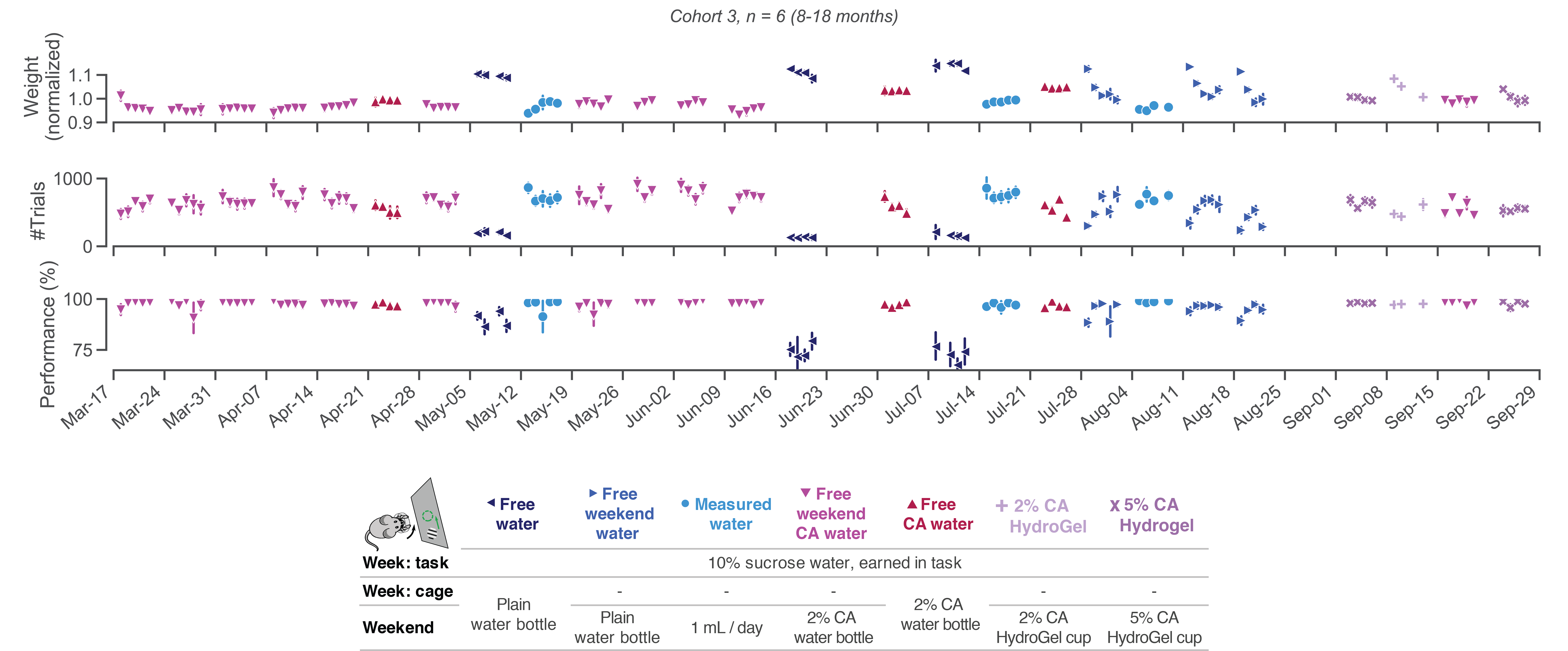

Supplement: Extended Data Figure 2-1 — Weight, trial counts and behavioral performance over time. Data as in Figure 2, shown over the full period of data collection. A leak in the rig tubing resulted in inaccurate reward volumes during two weeks of training (in late June and early September); these data were excluded from all analyses. Download Figure 2-1, TIF file. [file enu-eN-MNT-0230-20-s02.tif]

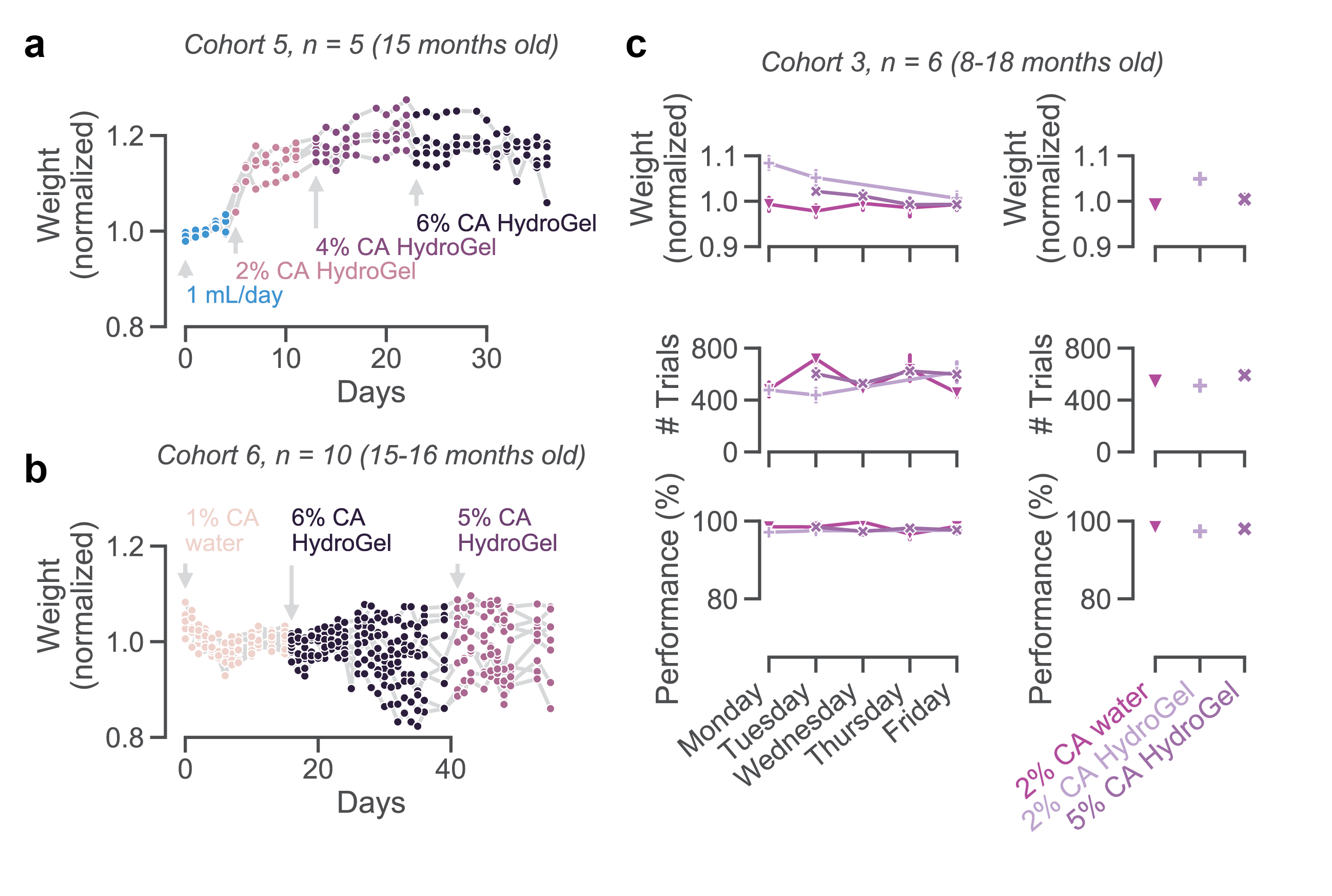

Supplement: Extended Data Figure 2-2 — CA can be dissolved in HydroGel instead of water. If bottled CA water cannot easily be provided (during travel, due to cage size restrictions, or when head implants preclude the use of bottle-top cages), CA can be mixed into HydroGel cups (https://www.clearh2o.com/product/hydrogel/) as an alternative to liquid water. HydroGel was melted by placing unopened 56-g cups in a 60°C oven until the gel had liquified (3–5 h). CA powder was then mixed into the liquefied gel and stirred thoroughly, before resealing the HydroGel cups and letting them solidify at 4°C. As the flavor and perceived aversiveness of CA may differ when dissolved in water or HydroGel, we again titrated CA concentrations to achieve stable animal weights. The observation that higher concentrations of CA are required in HydroGel to achieve the same behavioral effects agreed with informal human flavor perception of both substances. A, In a first cohort of animals (cohort 5: n = 5, 15 months), this required increasing concentrations from 1% to 6% m/v. Weight (from baseline on daily measured water), as animals were given free HydroGel with different concentrations of CA. B, With a second cohort of animals (cohort 6: n = 10, 15–16 months), switching from plain HydroGel to 6% CA HydroGel resulted in weights close to the institutional minimum of 80%. At 5% CA HydroGel, all animals showed stable weights. C, In our cohort of trained animals (Fig. 2), we confirmed that a weekend regime of 2% CA could be replaced by 5% CA in HydroGel. As in Figure 2, but comparing 2% CA water with 2% CA HydroGel and 5% CA HydroGel on weekends (same animals as shown in Fig. 2; see also Extended Data Fig. 2-1). Neither weekly trial counts (t(5) = –0.932, p = 0.3941, Bf10 = 0.522) nor performance on easy trials (t(5) = –1.444, p = 0.2083, Bf10 = 0.771) were significantly different in weeks following 2% CA water versus 5% CA HydroGel. Download Figure 2-2, TIF file. [file enu-eN-MNT-0230-20-s03.tif]

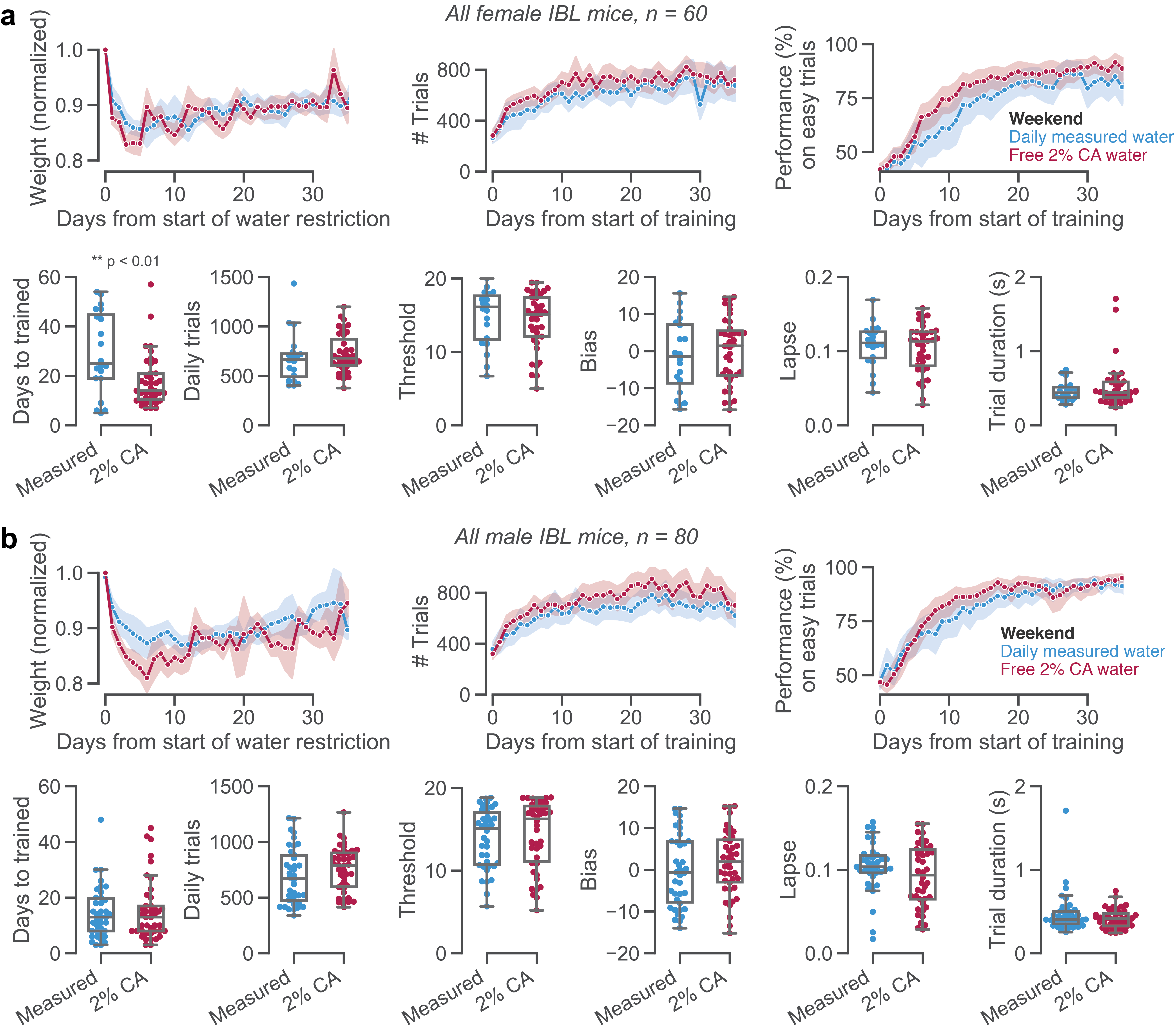

Supplement: Extended Data Figure 3-1 — Sex differences. We tested whether the weekend water regime (2% CA bottle vs measured water) differently affected (A) female and (B) male mice. Female mice given measured water on weekends learned the task slightly slower than female mice given 2% CA on weekends (t(27) = –2.89, p = 0.007, Bf10 = 7.731). This was not the case for male mice (t(77) = 0.30, p = 0.762, Bf10 = 0.242). Learning speeds showed a main effect of sex, and a significant interaction between water regime and sex (two-way ANOVA: effect of sex F(1) = 14.367, p < 0.001; effect of water regime F(1) = 4.645, p = 0.033; interaction F(1) = 9.457, p = 0.003). The overall slower learning speeds of female mice may be due to their lower weights, causing them to be satiated more quickly and performing fewer trials early in the training process (we gave all animals a fixed reward volume, independent of their body weight). We can speculate that animals’ weight and hydration balance may be slightly different in different water regimes, which interacts with motivation and learning speed in a sex-specific manner. Learning speeds differ between labs, which may be caused by various factors (The International Brain Laboratory et al., 2020). Further work is thus needed disentangle any sex differences in the effects of water regime on task learning. There was no significant effect of sex, or interaction between sex and water regime, for stable behavior upon training completion: daily trial counts (effect of sex F(1) = 0.345, p = 0.558; effect of water regime F(1) = 2.070, p = 0.153; interaction F(1) = 0.061, p = 0.806), visual threshold (effect of sex F(1) = 0.142, p = 0.707; effect of water regime F(1) = 0.002, p = 0.962; interaction F(1) = 0.584, p = 0.446), choice bias (effect of sex F(1) = 0.825, p = 0.365; effect of water regime F(1) = 1.374, p = 0.243; interaction F(1) = 0.027, p = 0.869), lapse rate (effect of sex F(1) = 2.085, p = 0.151; effect of water regime F(1) = 2.284, p = 0.133; interaction F(1) = 0.320, [file enu-eN-MNT-0230-20-s04.tif]

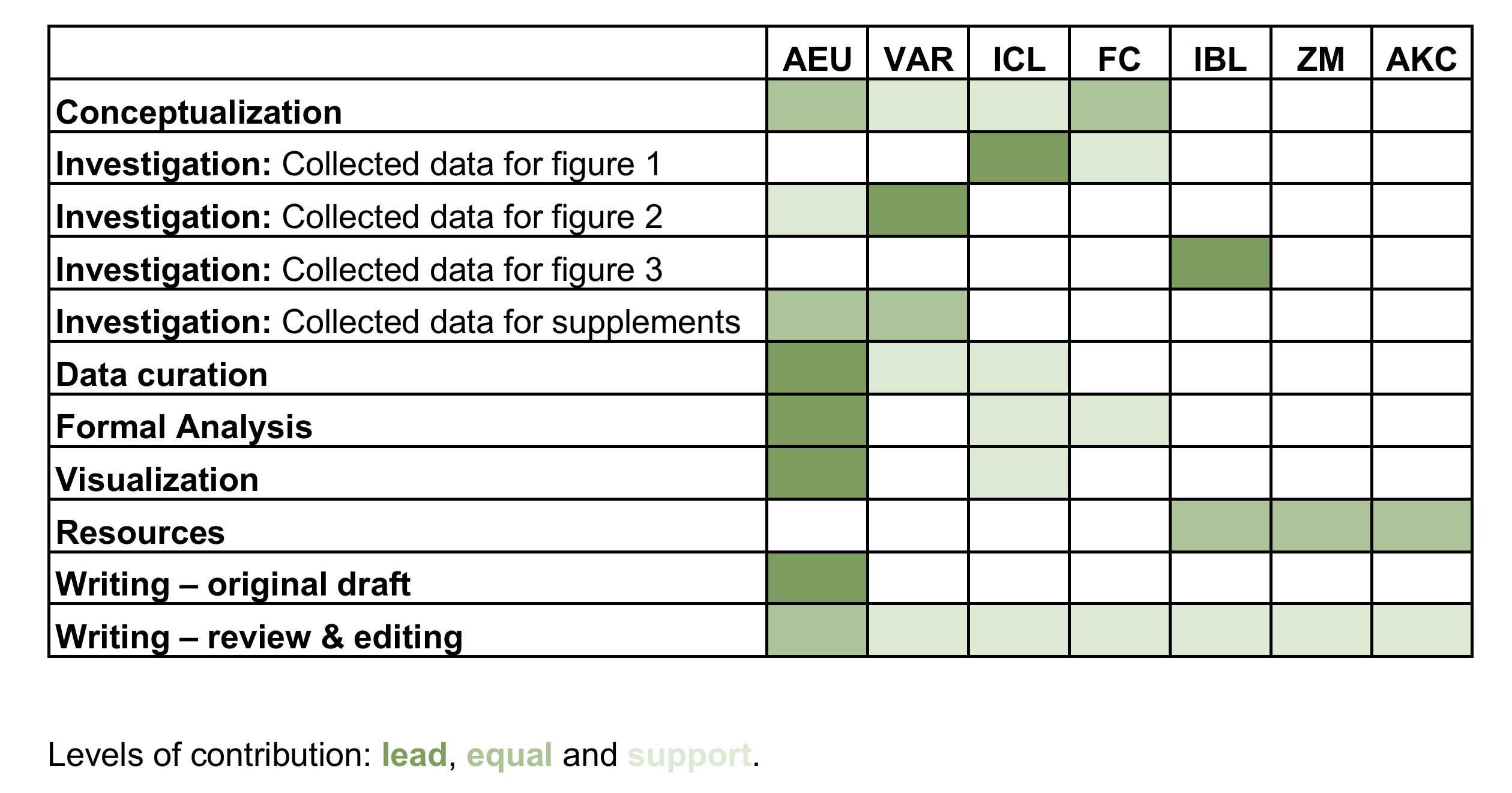

Supplement: Extended Data Ed1 — Contributions table. Download Extended Data Ed1, TIF file. [file enu-eN-MNT-0230-20-s06.tif]
